# Development of Thyroid Hormones and Synthetic Thyromimetics in Non-Alcoholic Fatty Liver Disease

**DOI:** 10.3390/ijms23031102

**Published:** 2022-01-20

**Authors:** Man Zhao, Huazhong Xie, Hao Shan, Zhihua Zheng, Guofeng Li, Min Li, Liang Hong

**Affiliations:** 1 Guangdong Key Laboratory of Chiral Molecule and Drug Discovery, School of Pharmaceutical Sciences, Sun Yat-Sen University, Guangzhou 510006, China; zhaom55@mail2.sysu.edu.cn (M.Z.); xiehzh6@mail2.sysu.edu.cn (H.X.); 17827063336@163.com (H.S.); zhengzhihualllleng@163.com (Z.Z.); 2 Health Science Centre, School of Pharmaceutical Sciences, Shenzhen University, Shenzhen 518060, China; liguofeng@mail.sysu.edu.cn

**Keywords:** NAFLD, NASH, thyroid hormones (THs), thyroid hormone receptor (TR), thyromimetics

## Abstract

Non-alcoholic fatty liver disease (NAFLD) is the fastest-growing liver disease in the world. Despite targeted agents which are needed to provide permanent benefits for patients with NAFLD, no drugs have been approved to treat NASH. Thyroid hormone is an important signaling molecule to maintain normal metabolism, and in vivo and vitro studies have shown that regulation of the 3,5,3’-triiodothyronine (T3)/ thyroid hormone receptor (TR) axis is beneficial not only for metabolic symptoms but also for the improvement of NAFLD and even for the repair of liver injury. However, the non-selective regulation of T3 to TR subtypes (TRα/TRβ) could cause unacceptable side effects represented by cardiotoxicity. To avoid deleterious effects, TRβ-selective thyromimetics were developed for NASH studies in recent decades. Herein, we will review the development of thyroid hormones and synthetic thyromimetics based on TR selectivity for NAFLD, and analyze the role of TR-targeted drugs for the treatment of NAFLD in the future.

## 1. Introduction

Non-alcoholic fatty liver disease (NAFLD) refers to the clinical syndrome of liver diseases caused by excessive deposition of fat in hepatocytes under non-alcoholic or other special liver damaging factors, including simple steatosis (SFL), non-alcoholic steatohepatitis (NASH), liver fibrosis, and cirrhosis [1,2]. With the continuous action of injury factors, simple steatosis will turn into NASH. Compared with simple steatosis, NASH is an important signal for the deterioration of the disease, and it will further develop into cirrhosis, even liver cancer if there is no regulation [3,4,5,6,7,8,9,10]. However, with a change of quality of life, the prevalence of NAFLD increases rapidly due to some dietary habits and lifestyle factors [11,12,13]. Currently, non-alcoholic steatohepatitis has become the fastest-growing cause of liver transplantation in the United States and is likely to become the largest cause in 2020 [14,15]. An epidemiological research involving more than 8.5 million people in 22 countries showed that NAFLD affects about 25% of the world’s population, with the highest reported prevalence in the Middle East (32%) and South America (30%), and the lowest in Africa (14%) [16]. It is worth noting that NAFLD not only develops rapidly in adults, but also has a great influence on children, and it is gradually becoming serious. Some surveys showed that the prevalence rate of NAFLD in children was close to 70–80% in obese individuals, and the ratio of male to female was 2:1 [17,18,19,20]. The above mentioned suggest a need for an effective treatment for NAFLD or NASH.

**Figure 1:**
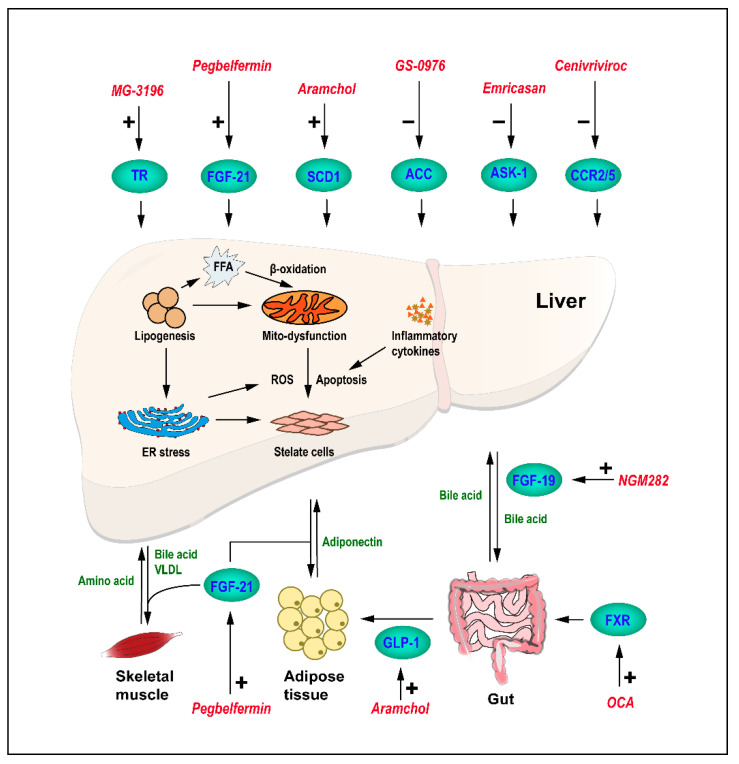
Representative therapeutic methods in the complex pathophysiology of NASH, which involved a variety of organs, tissues, and cells. Signals marked in green are protective against NASH, and those marked in red are current clinical trial drugs that ameliorated NASH as agonists/inhibitors (+/−) of various receptors marked in blue. Abbreviations: TR, thyroid hormone receptor; FGF, fibroblast growth factor; SCD1, stearoyl-CoA desaturase; ACC, acetyl-CoA carboxylase; ASK1, apoptosis signaling kinase 1; GLP-1, glucagon-like peptide 1; FXR, farnesoid X nuclear receptor; FFA, free fatty acids; and ROS, reactive oxygen species.

Given the serious threat to human life and health of NAFLD, in terms of prevention and control, it was recommended that people keep a good diet and living habits as far as possible, and many research results showed that diet and exercise, such as losing weight, reducing body fat, or keeping happy, and so on, could play a positive role to inhibit the development of NAFLD [13,21,22,23,24], but there is little benefit in patients with end-stage liver disease and it is difficult to get effective and lasting results by lifestyle intervention. Besides, some studies have confirmed that bariatric surgery could achieve good results, but this method is restricted by many taboos and only works in morbidly obese NASH patients [25,26,27]. Therefore, in recent decades, a large number of researchers and drug companies have made great efforts to develop drugs for NAFLD and NASH, trying to inhibit the NASH process. NAFLD is often associated with metabolic diseases (generally associated or secondary) such as cardiovascular disease, Type 2 diabetes mellitus (T2DM), and hypertension, of which pathological process often involved signaling pathways, such as oxidative stress, mitochondrial metabolism, or inflammation and intricately presented in the cellular, tissue, and organ levels, providing the basis for researchers to develop drugs with different targets [28,29,30,31,32,33] (Figure 1). For example: (I) Farnesoid X nuclear receptor (FXR) agonist Obeticholic acid (OCA) [34,35,36]; (II) Apoptosis signaling kinase 1 (ASK1) inhibitor Selonsertib [37,38]; (III) CCR2/CCR5 inhibitor Cenicriviroc [39]; (IV) Peroxisome proliferator-activated receptor (PPAR) agonists Rosiglitazone [40], Pioglitazone [41], and Elafibranor [42,43]; (V) Glucagon like peptide-1 (GLP-1) agonists Liraglutide [44]; (VI) Stearoyl-CoA desaturase (SCD1) Inhibitor Aramchol [45,46], (VII) PEGylated fibroblast growth factor 21 (FGF21) analogue (Pegbelfermin) [47], and (VIII) Thyroid hormone receptor (TR) drug Resmetirom [48], etc. Due to deleterious effects or other reasons leading to the studies of some drugs being stopped in the clinical stage, they were not used in the treatment of NASH eventually. 

For a long time, thyroid hormones (THs) have been considered an important signaling molecule to maintain normal metabolism of the body, such as lipid utilization, energy metabolism, and glucose metabolism, and were of great significance to tissue differentiation, growth, and maturation [49,50]. Therefore, abnormal thyroid hormone signaling is also associated with a variety of chronic diseases, including diabetes [51,52,53,54], cardiovascular disease [55,56,57,58,59,60,61], and liver-related diseases, especially nonalcoholic fatty liver disease (NAFLD) [62]. Of note, increasing evidence showed that THs have an obvious effect on ameliorating NAFLD under the thyroid hormone receptor (TR) which has an organ-targeting property [63,64,65]. As for TR drugs based on T3/TR axis as the favored targets for alleviating NASH, they have been constantly updated since the beginning of the research. The whole process will be reviewed below, and the current research advance and future direction will be analyzed.

## 2. Thyroid Hormones and Their Metabolites

The physiological effect of TH affects almost every organ and the liver is a crucial target organ, so the study of thyroid hormone and its metabolites are of great significance for the treatment of NAFLD or NASH.

### 2.1. T4/T3

**Figure 2:**
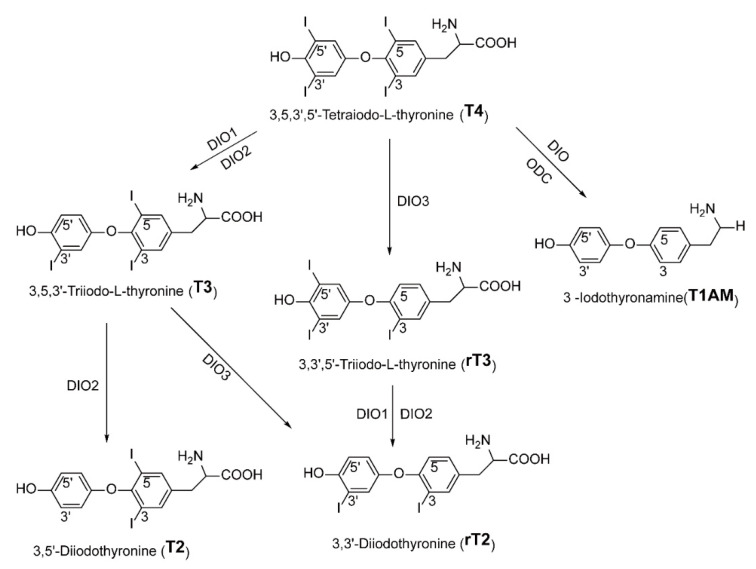
Metabolic pathways of thyroxine (T4). DIO isozymes catalyze reductive sequential removal of iodide from the phenolic. Abbreviations: DIO: iodothyronine deiodinase, and ODC: ornithine decarboxylase.

T4 (3,5,3′,5′-l-tetraiodothyronine) and T3 (3,5,3′-l-triiodothyronine), which usually represents the thyroid hormone, affect nearly every organ system. Thyroid hormone is produced through a feedback loop that includes the hypothalamus, pituitary, and thyroid gland, commonly referred to as the hypothalamic–pituitary–thyroid (H–P–T) axis [66,67,68]. The H–P–T axis involves a series of signal transduction cascades. The signals sent from the hypothalamus eventually reach the thyroid gland, triggering the release of thyroid hormones and performing balance regulation under the action of a series of deiodinases. T4 is the main secreted thyroid hormone, but it is much less active than T3. After entering the cell, it is usually transformed into an active T3 under the action of deiodinase 1/2 (DIO1/DIO2), and it can also be transformed into an inactive 3,3′,5′-l-triiodothyronine (reverse T3/ rT3) under the action of deiodinase 3 (DK = IO3). T3 and rT3 can be further deiodinated and transformed into T2 and rT2, respectively, to maintain the balance of thyroid hormone function [69,70,71] (Figure 2).

The role of T3 is mainly mediated by the thyroid hormone receptor (TR), which is a member of the nuclear receptor superfamily [72,73,74]. Its two receptor subtypes, TRα and TRβ, are encoded by *Thra* (also known as c-*erba*) and *Thrb*, respectively. Notably, TRα and TRβ exhibit conspicuous differences in expression pattern: The α isoform has a T3-binding splice product TRα1 and is highly expressed in the brain, heart, and skeletal muscles; TRβ has two major T3-binding splice products, and TRβ_1_ is predominately expressed in brain, liver, and kidney whereas the TRβ_2_ is limited to the hypothalamus, retina, and pituitary. In general, it can be recognized that TRα is mainly expressed in the heart, TRβ enriched mainly in the liver [75,76,77]. As mentioned above, the liver is one of the most important target organs of thyroid hormone, and the role of THs is mediated by TRβ in the liver. The expression of TH transporter, deiodinase, and TR in liver biopsy specimens of NASH patients at different stages was analyzed, and it was found that the expression of TR mRNA was negatively correlated with NASH score and further decreased with age [78], suggesting that T3 level in the liver plays an important role in NAFLD.

It has been found that T3 blocks the development of liver steatosis by increasing the β-oxidation of fatty acid by mitochondria and peroxidase, and decreasing liver-type fatty acid binding protein expression (l-FABP) in animal models [79], lacking of choline methionine diet (CMD) led to a large number of triglyceride accumulation in the liver and liver injury in mice, which is closely similar to the pathological and biochemical characteristics of human NASH and is used in the study of NASH commonly [80,81]. In fact, as an important regulator of metabolism in the body [49,50], T3 was initially considered as a key molecule causing hypothyroidism and hyperthyroidism and could regulate serum cholesterol and density lipoprotein [82,83,84]. As it can promote the metabolism of triglyceride, accelerate the decomposition of fat, and have a good effect on weight loss, T3 became one of the earliest anti-obesity drugs [85]. However, with the continuous discovery that TH signal changes in cells can lead to liver-related diseases, such as NAFLD and hepatocellular carcinoma (HCC), and even prevent the development of NAFLD [62,79], the focus of the study on THs/TR axis turn into NAFLD. However, an excess of thyroid hormone is associated with deleterious effects as the non-selective receptor binding, particularly on the heart (including tachycardia and sudden death) but also on bone and skeletal muscle [86]. Thus, the molecule selectively binding to the TRβ in the liver is needed to avoid serious side effects.

### 2.2. T2

T2 (3,5,-l-diiodothyronine), produced by T3 in the presence of deiodinase 2(DIO2) [87], is initially thought to be an endogenous metabolite that regulates T3 activity only. Interestingly, Mollica et al. found that T2 administration in high-fat diet (HFD) rats could significantly inhibit weight gain and had no effect on TSH level [88]. In fact, before this, analysis of in vitro and in vivo data by Ball and colleagues confirmed that T2 had significant thyroid-like activity, but with a 50–1000 times lower affinity to TR than T3 [89]. In liver sections of the rat model on a high-fat diet (HFD), T2 was found to reduce fat accumulation and T2 increased the oxidation rate of fatty acids and carnitine palmityl transferase activity at the mitochondrial level. Also, by stimulating mitochondrial uncoupling, the utilization efficiency of fatty acid substrates was reduced, and the oxidative stress of mitochondria was improved, and liver steatosis was significantly reversed in vivo finally [88]. In order to find out whether T2 reduces liver fat accumulation is a direct effect on the liver, Grasselli and co-workers studied the ability of T2 to reduce excess lipid in hepatocytes separated and treated with free fatty acid (FFA), and the results showed that added T2 or T3 in the FFA processing “fatty hepatocyte” could directly lead to the extra fat reduction of hepatocytes, by reducing hepatocellular fat accumulation related factors, which include: (i) lipid content and lipid droplet diameter; (ii) expression of PPAR-γ and PPAR-δ; and (iii) activities of CoA oxidase and antioxidant enzyme [90]. 

Since then, the role of T2 play anti-steatosis in vivo has been described in several NASH models, particularly in animal models. Among them, compared with the rats with HFD alone, the liver steatosis of the HFD rats treated with T2 completely disappeared, and the serum triglyceride and cholesterol levels reduced significantly, due to stimulating the activity of liver fatty acid oxidation and CPT, importantly, no change in heart rate was observed during or at the end of treatment [91]. Further research showed that the T2 worked by inhibiting insulin resistance rather than directly binding the TRβ, that is, directly acting on the liver nuclear protein SIRT1, which is a nuclear deacetylase regulating the liver gene expression under the interaction of TRβ and T3 [92,93], to adjust lipid metabolism and improve mitochondrial activity [94]. In addition to inducing oxidation pathways, T2 can also up-regulate the level of apolipoprotein B, stimulate lipoprotein secretion, and reduce hepatic excess fat [95]. Besides, both T2 and T3 can reduce hepatic triglyceride levels, induce autophagy and hepatic acylcarnitine flux strongly, while preventing the production of spindle-ceramide, which is known to contribute to oxidative stress in NASH. 

Overall, T2 shared positive effects of T3 on NAFLD, but there are differences in the role of T3 way [96]. Although serious heart weight side effects were found when high doses of T2 administration [97], a lot of experiment in vivo and in vitro suggested that the T2 could increase the basal metabolic rate and block fat accumulation without side effects of thyroid hormones [98], which indicated T2 was worthy of further research for prevention and cure of diseases, such as obesity and NAFLD [99].

### 2.3. T1AM 

The biogenic amine 3-iodothyronamine (T1AM) found in blood and tissues is another noteworthy thyroid hormone metabolite (THM). There is some controversy about its function since T1AM was first reported to be an endogenous thyroid hormone metabolite with antagonism to T3 [100]. As an endogenous THM, T1AM is an effective agonist for G-protein-coupled trace amine receptor TAAR1 in vitro. Application of T1AM in vivo can trigger severe hypothermia and bradycardia within minutes, and these significant effects on heart and metabolism are contrary to or different from the known functional parts of thyroid hormones [100]. Studies have shown that T1AM is mainly distributed in liver, brain, and muscle tissues, and no metabolic traces related to T1AM have been found in the heart [101]. Due to the high concentration of T1AM in the liver, it is speculated that the metabolic function of sugar and lipids occurs in the liver to some extent, which is also supported by some research results [102]. Chronic T1AM administration has been shown to induce transcriptional changes in hepatocytes and adipocytes. When rats were exposed to T1AM, the incidence of liver-related gene expression profile was significantly altered, manifested by increased lipid decomposition and oxidation [103]. In addition, in the polycystic ovary rat model, T1AM administration can induce a profound tissue-specific antilipogenic effect in liver and muscle by lowering gene expression of key regulators of lipid metabolism, including protein tyrosine phosphatase 1 B (PTP1B) that plays a major role in the intersection computes of insulin and lipid regulatory signaling pathways; and perilipin 2 (PLIN2) that regulates storage and hydrolysis of neutral lipids, leading to significantly reduce the intrahepatic triglyceride and cholesterol levels. It should be noted that since T1AM acts on TAAR1 rather than TR, it does not share similar cardiac side effects as T3, which is good information for the prevention and treatment of liver diseases such as NASH. Of course, although T1AM has an important effect on liver metabolism, numerous experiments in vivo are needed to confirm whether it can be used to treat NAFLD or HCC.

## 3. Synthetic Thyromimetics 

Thyroid hormone represented by T3, as an important molecule regulating lipid and glucose metabolism in the body [49,50], has been shown to play an obvious role in the prevention and treatment of NAFLD [62,79], but it is not used clinically due to its non-selective role, that may bring about serious side effects, such as increased heart rate and cardiac hypertrophy. Thyroid hormones acting on different thyroid hormone receptors (TRs) can produce different physiological effects [75,76,77,104]. In order to treat NAFLD or NASH without other organ side effects, the liver TRβ needs to be stimulated selectively, which leads to the structural design or modification of endogenous THs to meet receptor selectivity. Of course, the modified structure still needs to be repeatedly verified in experiments, which is usually not a smooth process. It has taken decades from the initial thyroid hormone drug GC-1 [105] to the most likely successful MGL-3196 still in clinical trials [106], during which it has experienced many trials and failures (Table 1).

### 3.1. GC-1, GC-24 

GC-1 (3,5-Dimethyl-4 (4′-hydroxy-3′-isopropylbenzyl)-phenoxy) acetic acid), also known as Sobetirome, is the first synthetic TR selective thyroid hormone drug. Based on the structure of endogenous thyroid hormone T3, the designer replaced three iodine with methyl and isopropyl, replaced diaryl ether with methylene, and replaced the amino acid side chain, which ultimately makes synthesis easier [105]. Besides, GC-1 has similar receptor selectivity for TRβ1 comparing to T3, but its affinity for TRα1 is nearly 10-fold lower than T3, and enhancing liver TR targeting [105]. The TRβ selectivity of GC-1 was confirmed in follow-up studies: Trost et al. analyzed the effects of GC-1 and T3 as a control in rats with hypothyroidism. The results show that equal dose administration, compared with the T3, GC-1 reduces triglyceride better and reduce cholesterol similarly, and no accelerated heart rate like T3 caused was observed [107]. Grover et al. evaluated the selectivity of GC-1 in lipid lowering and metabolic rate changes relative to tachycardia. After drug delivery on cholesterol feeding rats for seven days, they found that the effectiveness of GC-1 for reducing cholesterol (ED5: 190 nmol/kg/D) higher about 30 folds than induce tachycardia (ED50: 5451 nmol/kg/D), but T3 not, and compared with the T3, GC-1 improve metabolic rate (ED50: 477 nmol/kg/D) selectively about 10-fold related to the tachycardia [108].

In terms of liver diseases, Perra et al., employing the CMD-diet model, confirmed that the therapeutic effect of GC-1 on NAFLD was consistent with that of T3, such as preventing the occurrence of liver steatosis, promoted the decomposition of existing fat accumulation, and reduced liver triglyceride with a concomitant decrease of lipoperoxidation [79]. GC-1 is not only effective in various nutritional models [109,110], long-term administration of GC-1 during liver tumorigenesis, can also completely eliminate the hepatic pre-neoplastic lesions. In addition, a recent study on hepatocellular carcinoma (HCC) has shown that GC-1 significantly inhibits HCC progression without affecting β-catenin and its downstream targets during anti-tumoral effects [111,112,113]. Besides, other studies showed that GC-1 also shared a proliferation promotion effect of T3, which increases the proliferation of hepatocytes significantly in pre-damaged liver tissues [114,115,116]. These results indicate the future of GC-1 in liver disease. But it is worth noting that previous studies also found that the GC-1 administration, at the same time of improving liver disease model, leads to fasting blood sugar and insulin resistance to the model rats, prompted the complex relationship between TR activation and insulin sensitivity, which indicated the importance of considering the adverse effects due to insulin sensitivity when noticing the TR agonists which may be valuable in the treatment of NAFLD [109,110].

GC-24, a second-generation TR-selective agonist, is a derivative of GC-1 further modified [117]. The structural feature of GC-24 is that a benzyl group replaces 3′-iodine, and Borngraeber et al. observed that the introduction of this large hydrophobic group is critical to improve the receptor selectivity, leading to the binding affinity of GC-24 to TRβ 40-fold higher than that of TRα. In the HFD rats model, however, GC-24 made a significant improvement in reducing plasma triglycerides, body fat, glucose tolerance, and insulin sensitivity without affecting the weight of the heart, but it cannot restore hypercholesterolemia, increased hepatic cholesterol content, elevated non-esterified free fatty acids (NEFA), and IL6 levels, suggesting that these metabolic pathways are low sensitivity for activated-TRβ during a high-fat diet [118]. Studies also showed that GC-24 had poor liver target than GC-1 or T3 [119], which limited the development of GC-24 in NASH.

### 3.2. KB141, KB2115 

**Figure 3:**
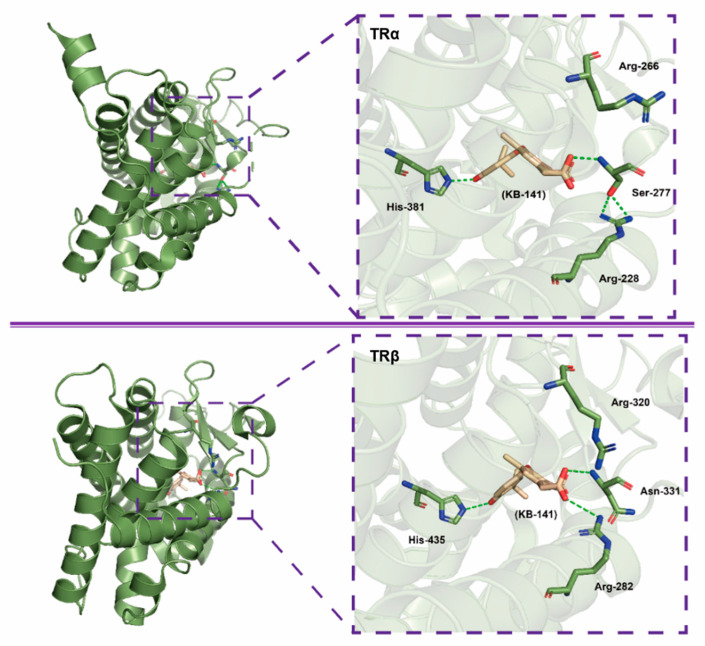
Interaction of the ligand KB-141 with TRα (upper) and TRβ (lower) as seen in the crystallographic structures of the LBD/ ligand complexes. Carbon atoms of LBD are depicted as bottle green, oxygen as red, nitrogen as blue, and hydrogen bonding interactions are represented as dashed green lines. PDB code: 1NAV and 1NAX [120].

At the same time, another TR-selective molecule KB141 (3,5-dichloro-4-(4-isopropylacetic acid)) has also been synthesized, with the structure of GC-1, of which 3,5-digit methyl were replaced by two chlorine atoms [120]. KB141 has a 10-fold higher affinity to TRβ than TRα and was found that the ligand-binding pocket was buried deep within the receptor by analyzing the eutectic of the ligand with TRβ or TRα, and the difference of binding site only in one amino acid residue (the Asn331 in TRβ1 replaced by the Ser277 in TR α1) [117,120] (Figure 3), leading to the difficulty of thyroid hormones structural modification for improving TRβ selectivity. In mice, rats, and monkeys, KB141 reduces cholesterol, lipoprotein, and weight significantly by enhancing metabolism and also reduces triglyceride and free fatty acids of the liver without effect on the heart, showing that KB141 is a very potential anti-obesity agent. Unfortunately, there is no application in NAFLD and human clinical trials [121,122,123,124].

Subsequently, a brominated TR-selective thyroid hormone replacement drug, KB2115 (3-(3,5-dibromo-4-(4-hydroxy-3-(1-methylethyl)-phenoxy)-phenyl)-amino-3-oxopropanoic acid), has been reported to be more liver targeting than T3 in terms of organ intake [125]. In moderately overweight and hypercholesterolemia subjects, KB2115 was found to be safe and well tolerated, with a 40% reduction in total and low-density lipoprotein cholesterol (LDL-C) after administration of two weeks. Besides, the synthesis of bile acids was stimulated without evidence of cholesterol increase, suggesting that KB2115 induces net cholesterol excretion. Importantly, KB2115 has no detectable effect on the heart, which is beneficial for further human trials [125]. In a 12-week phase II clinical trial, KB211 reduced LDL-C, triglyceride, and atherosclerotic lipoprotein levels in patients treated with statins [126]. In order to determine the effectiveness of KB2115 for NAFLD and hepatic insulin resistance, the therapeutic effects of HFD in male Sprague–Dawley rats were evaluated. Similar to GC-1, KB2115 effectively prevented liver steatosis in this animal model, and fasting hyperglycemia did not occur, although the disadvantages of increased fasting blood insulin were preserved [109]. In addition, KB2115 can also reduce the burden of liver steatosis in *ob/ob* mice and significantly reduce the liver triglyceride level [110]. Notably, a recent study found that KB2115 has the same hepatogenic activity as T3 and GC-1 in the absence of obvious hepatotoxicity, suggesting that it can be used for regenerative therapy in liver transplantation or other surgical procedures [127]. Unfortunately, there were adverse effects on dogs’ cartilage when KB2115 was discontinued after long-term administration, resulting in the direct termination of phase III clinical trials of KB2115.

### 3.3. MB07811(VK2809), MB07344 

Then, there was a TR-selective agonist with phosphonic acid: (2R,4S)-4-(3-chlorophenyl)-2-((3,5-dimethyl-4-(4′-hydroxy-3′-isopropylbenzyl) phenoxy) methyl)-2-oxido-(1,3,2)-dioxaphosphonane (MB07811, known as VK2809) grabbed attention. As a prodrug, after first-pass hepatic extraction, it cleaved, catalyzed by the CYP3A of the Cytochrome P450 enzymes, to negatively charged TR agonist (3,5-dimethyl-4-(4′-hydroxy-3′-isopropylbenzyl) phenoxy) methylphosphonic acid (MB07344) [128]. The remarkable influence of TR-responsive genes expression in liver and extrahepatic tissues, compared with T3 and liver-targeted agonist KB141, revealed that MB07811 has a better liver-targeted property, and significantly reduced cholesterol and serum and liver triglycerides in a dose without affecting body weight, blood glucose, etc., in a mice model of diet-induced obesity [128]. Further research showed MB07811 dramatically reduced free fatty acid (FFA) and triglyceride and ameliorates steatosis, by contrast, T3 induced adipocyte lipolysis, and the ability of the hepatic steatosis was attenuated in vivo and in vitro, indicating the peripheral FFA influx of liver may be partially offset the T3 steatosis activity [129]. Cable et al. also suggested that the activation of TR in the liver by MB07811 would lead to increase of liver–mitochondrial respiration rate, plasma acylcarnitine levels, and a differential expression of liver gene, thus clearing liver lipids. It should be noted that long-term treatment with MB07811 does not result in liver fibrosis or other histological liver damage and does not increase heart weight or decrease the expression of pituitary thyrotrophic hormone (TSH) [129].

MB07811 represents a new class of liver targeted TR agonists to a certain extent, and experimental results also suggested its therapeutic benefits for hyperlipidemia patients with NAFLD [130]. Most recently, a study has found that MB07811 reduced liver steatosis in the glycogen storage disease Ia (GSD Ia). GSD Ia (known as von Gierke’s disease) could lead to increase levels of glycogen and triglycerides in the liver. Patients with increased glycogen and triglyceride levels in the liver are apt to develop steatohepatitis, cirrhosis, and have an increased risk of hepatocellular adenoma and cancer. MB07811 decreased liver mass and triglyceride content in GSD Ia mice, and research suggested MB07811 play a role through promoting mitochondrial oxidation by stimulating hepatic autophagy flux, such as increased LC3B-II and reduced p62 protein levels, and by increasing CPT1 and FGF21 expression and mitochondrial biogenesis [131], which also demonstrated a potential therapy for thyroid-like hormone drugs. Interestingly, MB07811, which has been stagnant since phase I clinical trials, was entered phase II and renamed as VK2809 [132].

### 3.4. MGL-3196 

Recently, a new liver-directed and TR-selective agonist 2-(3,5-dichloro-4-(5-isopropyl-6-oxo-1,6-dihydropyridazin-3-yloxy) phenyl)-3,5-dioxo-2,3,4,5-tetrahydro (1,2,4) triazine-6-carbonitrile, known as MGL-3196 (also known as Resmetirom), has been synthesized. The introduction of cyanoazauracil substituent significantly improved the selectivity and efficacy of MGL-3196. A functional coactivator recruitment assay in vitro, MGL-3196 showed a 28-fold higher selectivity for TRβ than TRα. In the rat heart model, there was no cardiac adverse effect and it was shown to be safe for the central thyroid axis. It should be noted that MGL-3196 not only promoted the reduction of cholesterol but also reduced the size of the liver, which was second only to the reduction of triglycerides but had no effect on the heart, kidney, and bone [133]. The safety MGL-3196 was assessed in 53 healthy volunteers. When the oral dose of 50 mg or higher was given once daily for two weeks, the subjects had no adverse reactions and good tolerance, and the favorable results of LDL-C and triglyceride (TG) were also observed [134]. These excellent results suggested the strong potential of MGL-3196 in the treatment of NAFLD, which has been demonstrated in the later clinical trials. In phase II clinical trials, MGL-3196 had good effects on plasma lipids compared with placebo: LDL-C was reduced by 22.3%; triglyceride was reduced by 30.8%; lipoprotein (a) had a 37.9% lower lipid, and adverse events are mild, even a moderate adverse event to keep a balance between groups such as the increased incidence of mild temporary diarrhea caused, but not significantly affects for the TSH levels, bone mineral density, cardiovascular or the insulin sensitivity [135]. In addition to significantly reducing liver fat content in NASH patients, MGL-3196 can also reduce atherosclerotic lipoprotein particles [48,136]. The latest result of MAESTRO Phase III NASH clinical studies (NCT03900429) showed that: (i) the liver fat reduction as MGL-3196 administration for three months had a clear predictive capacity for NASH resolution and fibrosis reduction on subsequent liver biopsy; (ii) once-daily oral 80 mg and 100 mg Phase III doses of MGL-3196 deliver leading to the reductions of 50% at least and over 60% reductions in liver fat, respectively, even a reduction of fibrosis (>60%), and (iii) MGL-3196 significantly (*p* < 0.001) reduced markers of net collagen deposition in the liver, which was supporting the anti-fibrotic action of MGL-3196 [137].

## 4. Conclusions and Perspective 

Nonalcoholic fatty liver disease (NAFLD) is a series of diseases characterized by excessive accumulation of fat in the liver without the influence of alcohol. Nonalcoholic steatohepatitis (NASH) has attracted the most attention because it is more likely to progress to advanced liver disease and has become the most common reason for liver transplantation today [10]. Although improving lifestyle and diet could obtain significant effects in NASH, medication is necessary for patients with severe conditions [24], and there are no FDA-approved drugs to treat NASH. The growing understanding of the mechanisms and pathways of NASH has accelerated the development of numerous medical approaches to NASH for inhibiting or even reversing the progress of NASH in different ways. Besides, studies have shown that gut microbiota affects NAFLD by influencing intestinal permeability and the generation of short-chain fatty acids and ethanol [138]. Furthermore, cyclophilin inhibition [139] and IL-1 inhibitors [140] also play an important role in NASH prevention. Above these provide some new research directions for treating NASH, but instead of directly targeting the liver to alleviate NASH, they often alter NAFLD-related indicators, such as free fatty acids, triglycerides, and density lipoproteins in other ways, which may pose a risk of post-administration side effects, such as compensatory changes in other indicators. In addition, new therapeutic approaches, such as intestinal flora, need to be further confirmed in the mechanism of efficacy and possible adverse effects.

The modification of liver-direct thyroid hormone receptor agonist has been experienced decades, based on the endogenous thyroid hormone T3 structural skeleton, for increasing the receptor selectivity to reduce the side effects. However, with regard to TRα and TRβ, of which amino acid sequence of ligand binding domain (LBD) was about 75% identical, and the active sites of the hydrophobic pocket differ by only a single amino acid (Asn-331 in TRβ versus Ser-277 in TRα) [117,120], which is the maximum limit of the T3-based structure modification with high affinity difference of the TRs. Of course, there are other pharmacological strategies besides selective action on TR directly, for example, the use of thyroid hormones with low iodine content (e.g., T2), which suggests the potential of deiodinase as a novel target at the same time. And the use of prodrug principles (e.g., VK2809) leads to reducing the systemic distribution of agents. In addition, the function of VK2809 involves the activation of the autophagy pathway to enhance metabolism, which is also worthy of consideration [141].

Actually, we have always hoped to find a more efficient method to accurately screen small molecules with high TRβ receptor selectivity. In the past, the development of TR drugs was based on the modification of the original binding pocket and the molecular framework of T3 to improve the selectivity of receptor subtypes. However, it is difficult to completely eliminate the undesirable effects as the selectivity of receptor subtypes. We wonder whether we can get rid of inherent thinking and find a specific binding pocket of TRβ subtype which has an equivalent biological function of ameliorating NASH like T3 but having no influence on another organ, and which just needs a small molecule ligand with a new skeleton. This requires large-scale small molecule screening and it is difficult to obtain desirable results only through computer virtual docking in this case. Instead, the other methods of screening small molecules that can selectively bind TRβ at the protein level should be considered, such as surface plasmon resonance (SPR) and other technologies [142]. In this way, it is possible to discover new binding modes of a small molecule with TR receptor protein, and then verify the screening results through further physiological activity data. Of course, in order to carry out TR drugs more conveniently and quickly, there is still an urgent problem that needs to be solved, which is that there is no specific indicator to prove the activation of TR and improvement of NASH. This is also a major obstacle to the development of new small molecules and requires the joint efforts of scientists in the field of NASH.

For the moment, MGL-3196 is the most promising TRβ-selective agonist for NASH treatment and it obtained excellent results in clinical trials, such as reducing plasma LDL-C and triglyceride concentrations in NASH patients accompanied by a cardiovascular disease with cardiac protective potential, but it necessitates the assessment of safety based on more data of phase III clinical trials, especially in the area of underlying heart and skeletal toxicity. On the other hand, VK2809 is in phase II clinical trials as well and has great potential. In a nutshell, the TR-selective drug development is continuing. With regard to the treatment of NASH, the experts agree that combination therapy is the future of NASH treatment, and the accumulation of lipotoxic lipids within the liver can powerfully drive NASH and fibrosis development, thus it is necessary for the successful combination that contains a potent antisteatotic drug [106]. As a result, those drugs which were unsuccessful in NASH therapies previously will have potential, and, of course, MGL-3196 is already one of the most attractive NASH drugs in terms of combination therapy.

## Figures and Tables

**Table 1:** Summary of synthetic thyromimetics.

Compounds	Structure	Beneficial Effects	Deleterious Effects	Clinical Trials
Sobetirome (GC-1)	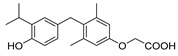	(1) 10-fold lower affinity of TRα (2) Reducing cholesterol level (3) Inhibiting HCC development (4) Liver regeneration	Fasting blood sugar and insulin resistance	Ending in phase I
GC-24	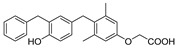	(1) 40-fold higher affinity of TRβ (2) Lower insulin sensitivity	(1) Low sensitivity for activated-TRβ (2) No hepatic targeting	——
KB-141	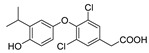	(1) Metabolic enhancement (2) Weight loss	——	——
Eprotirome (KB2115)	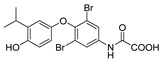	(1) Reducing triglycerides level markedly (2) Liver targeting (3) Liver regeneration	(1) Increasing fasting blood insulin (2) Adverse effects on dogs’ cartilage of withdrawal	Ending in phase III
M07811 (VK2809) /MB07344	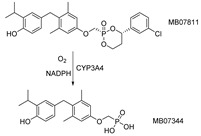	(1) Reducing cholesterol and triglycerides level (2) Inhibiting hepatic steatosis (3) Promoting hepatocyte proliferation	——	Phase II ongoing
Resmetirom (MGL-3196)	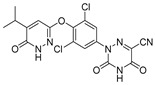	(1) Reducing cholesterol and triglycerides level (2) Inhibiting hepatic steatosis and fibrosis (3) Reducing hepatic fat markedly (4) Heart protection	——	Phase III ongoing

## Abbreviations

NAFLD: Non-alcoholic fatty liver disease; NASH, nonalcoholic steatohepatitis; HCC, hepatocellular carcinoma; TR, thyroid hormone receptor; T4, tetraiodothyronine; T3, triiodothyronine; T2, diiodothyronine; T1AM, 3-iodothyronamine; DIO, deiodinase; FGF, fibroblast growth factor; SCD1, stearoyl-CoA desaturase; ACC, acetyl-CoA carboxylase; ASK1, apoptosis signaling kinase 1; GLP-1, glucagon-like peptide 1; FXR, farnesoid X nuclear receptor; FFA, free fatty acids; ROS, reactive oxygen species; PTP1B, protein tyrosine phosphatase 1 B; PLIN2, perilipin 2; LDL-C, low-density lipoprotein cholesterol; HFD, high-fat diet; SPR, surface plasmon resonance; and LBD, ligand binding domain.
